# An Experimental Model of Neuromyelitis Optica Spectrum Disorder–Optic Neuritis: Insights Into Disease Mechanisms

**DOI:** 10.3389/fneur.2021.703249

**Published:** 2021-07-23

**Authors:** Sofie Forsberg Soerensen, Martin Wirenfeldt, Agnieszka Wlodarczyk, Marlene Thorsen Moerch, Reza Khorooshi, Dina S. Arengoth, Soeren Thue Lillevang, Trevor Owens, Nasrin Asgari

**Affiliations:** ^1^Department of Neurobiology, Institute of Molecular Medicine, University of Southern Denmark, Odense, Denmark; ^2^Department of Pathology, Odense University Hospital, Odense, Denmark; ^3^Department of Clinical Immunology, Odense University Hospital, Odense, Denmark; ^4^Department of Neurology, Slagelse Hospital, Slagelse, Denmark; ^5^Institute of Regional Health Research, University of Southern Denmark, Odense, Denmark

**Keywords:** optic neuritis, aquaporin-4 immunoglobulin G, antibody-mediated, type I interferon (IFN), disease model animal

## Abstract

**Background:** Optic neuritis (ON) is a common inflammatory optic neuropathy, which often occurs in neuromyelitis optica spectrum disease (NMOSD). An experimental model of NMOSD-ON may provide insight into disease mechanisms.

**Objective:** To examine the pathogenicity of autoantibodies targeting the astrocyte water channel aquaporin-4 [aquaporin-4 (AQP4)-immunoglobulin G (AQP4-IgG)] in the optic nerve.

**Materials and Methods:** Purified IgG from an AQP4-IgG-positive NMOSD-ON patient was together with human complement (C) given to wild-type (WT) and type I interferon (IFN) receptor-deficient mice (IFNAR1-KO) as two consecutive intrathecal injections into cerebrospinal fluid *via* cisterna magna. The optic nerves were isolated, embedded in paraffin, cut for histological examination, and scored semi-quantitatively in a blinded fashion. In addition, optic nerves were processed to determine selected gene expression by quantitative real-time PCR.

**Results:** Intrathecal injection of AQP4-IgG+C induced astrocyte pathology in the optic nerve with loss of staining for AQP4 and glial fibrillary acidic protein (GFAP), deposition of C, and demyelination, as well as upregulation of gene expression for interferon regulatory factor-7 (IRF7) and CXCL10. Such pathology was not seen in IFNAR1-KO mice nor in control mice.

**Conclusion:** We describe induction of ON in an animal model for NMOSD and show a requirement for type I IFN signaling in the disease process.

## Introduction

Optic neuritis (ON) is a common inflammatory demyelinating condition of the optic nerve (1) that is highly associated with neuromyelitis optica spectrum disorder (NMOSD) (2) and multiple sclerosis (MS) (3, 4). NMOSD is an autoimmune astrocytopathy, where the damage to astrocytes exceeds the damage to myelin and neurons, so differing from MS (4–6). ON related to NMOSD (NMOSD-ON) is more likely to cause recurrent episodes and worse visual outcome than ON related to MS (7). NMOSD is associated with antibodies directed against the water channel aquaporin-4 (AQP4), which is expressed on astrocytes in the central nervous system (CNS), including the optic nerve and on retinal Müller cells (8, 9). AQP4-immunoglobulin-G (AQP4-IgG) mediates pathogenesis by binding selectively to AQP4 on CNS astrocytes, leading to complement (C)-dependent astrocyte injury and inflammation (10).

Data concerning optic nerve pathology after AQP4-IgG exposure are limited, and the predominant involvement of the optic nerve in NMOSD still lacks a mechanistic explanation. This may be provided by an animal model of antibody-mediated ON. A number of studies have reported induction of NMOSD-like histopathology in experimental animals (11–13). In line with others, our group has previously evaluated the pathogenic impact of AQP4-IgG in the cerebrospinal fluid (CSF) and reported that intrathecal injection of AQP4-IgG together with human C into the CSF or brain striatum was sufficient to induce NMOSD-like pathology in the brain of naive mice (14–16). The pathology was dependent on type I interferon (IFN) response (16, 17). We have reported NMOSD-like lesions in various areas of the brain, cerebellum, brain stem, and periventricular areas following intrathecal administration (14). In the current investigation, we studied optic nerve-specific disease mechanisms and examined whether AQP4-IgG administered together with C as intrathecal injections into the CSF reached the optic nerve and induced NMOSD pathology in the optic nerve.

## Materials and Methods

### Experimental Design

**Figure 1:**
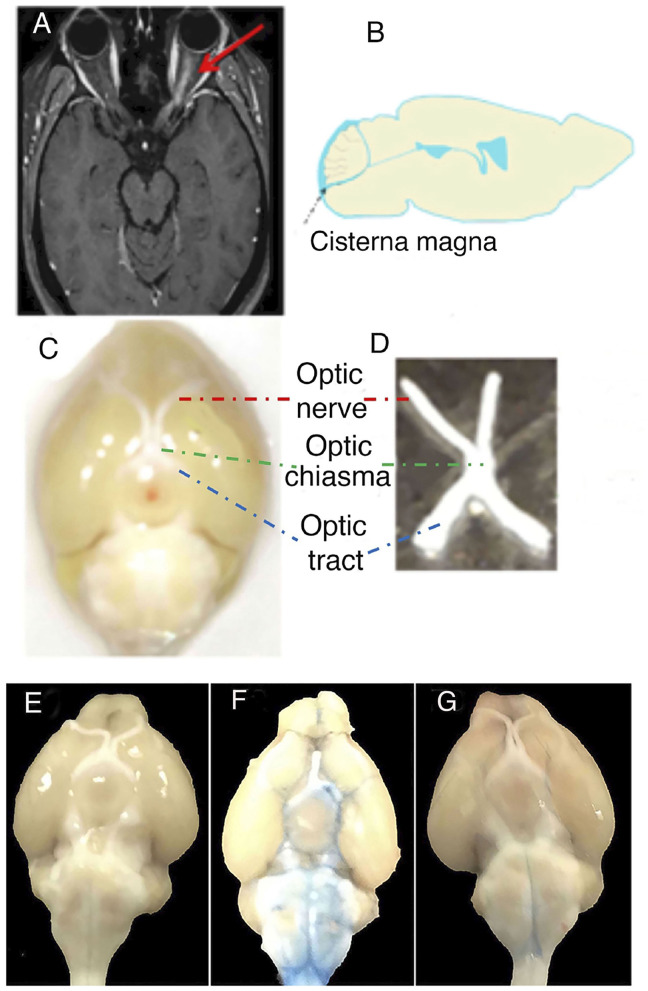
Translational and experimental design on optic neuritis (ON). **(A)** The figure shows the MRI of a patient with neuromyelitis optica spectrum disorder (NMOSD), seropositive for anti-aquaporin 4 antibodies [aquaporin-4-immunoglobulin G (AQP4-IgG)], with gadolinium-enhanced T1-weighted image showing ON on the left side. **(B)** Sagittal section of mouse brain indicating how NMOSD-ON patient-derived IgG was injected intrathecally into the cerebrospinal fluid *via* cisterna magna. **(C)** Anatomical structures of the anterior visual system in the mouse brain. The anatomical structures include the optic nerve (red), optic tract (blue), and optic chiasma (green). **(D)** Optic nerve including optic chiasma isolated from mouse. Original magnification × 10 **(C)**. **(E–G)** The tracer Evans blue dye in a diffusion study reached the optic nerve in the cerebrospinal fluid by intrathecal injection into cisterna magna. **(E)** Unmanipulated, **(F)** Evans blue dye diffusion at 0.5 h after intrathecal injection, and **(G)** Evans blue dye diffusion at 48 h after intrathecal injection.

The experimental design was based on intrathecal administration of IgG as previously reported (14, 15). The optic nerve was studied, and IgG from an AQP4-IgG-positive NMOSD-ON patient or normal human IgG from a healthy control, together with pooled normal serum as a source of C, was administered to mice as two consecutive intrathecal injections 24 h apart into the CSF (*via* cisterna magna) (18, 19) (Figures 1A–D).

Quantitative real-time PCR (RT-qPCR) and histological analyses were performed 2 and 4 days after the first intrathecal injection, respectively.

#### Mice

Adult female C57BL/6 [wild-type (WT)] mice were purchased from Taconic (Taconic, Denmark), and adult female type I IFN receptor-deficient mice (IFNAR1-KO) on a C57BL/6 background were bred from mice originally provided by Prof. Marco Prinz (20). The mice entered experiments at the age of 8–10 weeks with weights between 15 and 22 g as described previously (17). The mice were housed according to standard operating procedures of the Biomedical Laboratory, University of Southern Denmark. All experiments were conducted in accordance with the Danish Animal Experiments Inspectorate (approval number 2020-15-0201-00652). The mice were assessed by measurement of whole-body weight and gross evaluation of well-being. Assessment of behavioral or motor changes was not part of the study design. The weight of the animals did not differ between mice receiving AQP4-IgG and controls.

#### Human Immunoglobulin G and Complement

IgG was purified from an NMOSD patient who participated in a Danish cohort study (21). The measurement and purification of IgG are described elsewhere (14, 22). C originated from a pool of healthy serum donors (14). The use of human material was approved by the Biomedical Research Ethics for the Region of Southern Denmark (ref. no. S-20080142).

#### Intrathecal Injections

Mice were anesthetized using isoflurane inhalation (ScanVet, Denmark), and the back of the head was shaved. Just before the intrathecal injection, mice received a subcutaneous injection of buprenorphine (Temgesic®, 0.1 mg/kg of body weight, Indivior UK Ltd., UK). A 30-gauge needle (bent at 55°, 2–2.5 mm from the tip) (Covidien, USA) attached to a 50-μl Hamilton syringe (Hamilton Bonaduz AG, Switzerland) was inserted at the midline in the cleft between the atlas and occiput into the intrathecal space of the cisterna magna. Mice were injected with 300 μg AQP4-IgG together with 288 μg C in a total volume of 10 μl every 24 h for two consecutive days. The study included 41 WT [unmanipulated (*n* = 11), AQP4-IgG+C (*n* = 20), normal-IgG+C (*n* = 10)] and nine IFNAR1-KO mice [unmanipulated (*n* = 3), AQP4-IgG+C (*n* = 3), normal-IgG+C (*n* = 3)] for histology. These studies were performed over six separate experiments. In addition, six WT mice included in the tracer experiment and 15 mice were included for qPCR analysis [unmanipulated (*n* = 4), receiving AQP4-IgG+C (*n* = 5), normal-IgG+C (*n* = 6)]. A total of five mice were excluded due to lack of revival after anesthesia (*n* = 2) or loss of tissue in processing (*n* = 3). Control mice included mice that received a similar concentration and volume of normal IgG and C as well as unmanipulated mice. For tracer experiments, mice were intrathecally injected with 10 μl 0.1% Evans blue (Sigma Aldrich, Germany).

#### Optic Nerve Processing

Mice were sacrificed 2 or 4 days after the first intrathecal injection by an overdose of sodium pentobarbital (Euthanimal®, 200 mg/kg of body weight, Glostrup sygehusapotek, Denmark) and intracardially perfused with 20 ml ice-cold phosphate-buffered saline (PBS). Optic nerves were isolated in one piece and fixed in 4% paraformaldehyde (Sigma-Aldrich, Denmark) in PBS or 4% neutral buffered formalin (CellPath, Powys, UK) for 4–24 h at 4°C. Optic nerves were dehydrated and embedded in paraffin using a VIP 1,000 tissue processor (Sakura, Torrance, USA) or an automatic HMP 110 tissue processor (Microm UK Ltd., UK). Paraffin-embedded optic nerves were cast into blocks and cut into 4- or 6-μm-thick sections using a Thermo Shandon Finesse ME microtome (Thermo Shandon Ltd., Thermo Fischer Scientific, UK) or a Leica RM2255 Fully Automated Rotary Microtome (Leica Biosystems, Buffalo Grove, USA). Each section was placed on a water-filled paraffin stretch bath (TFB 35, Medite, Germany) at a temperature of 45°C, collected on microscope slides (superfrost® plus, Thermo Fisher Scientific, Germany, or FLEX IHC Microscope slides, Dako/Agilent, Glostrup, Denmark), and dried overnight. The following day, sections were incubated for 1 h at 60°C and stored at 4°C until use.

For examination of gene expression levels, optic nerves were stored immediately after isolation in 350 μl RLT mixture [10 μl β-mercaptoethanol (Sigma-Aldrich, France) per 1 ml RLT buffer (Qiagen, Germany)] at −80°C. For the tracer experiment, mice were sacrificed by an overdose of sodium pentobarbital and intracardially perfused 0.5, 1, 12, 24, or 48 h after injection with Evans blue.

#### Histopathological Evaluation of Optic Nerves

Histopathology analysis was performed 4 days after the first intrathecal injection. Evaluation of histological changes was performed semi quantitatively. Based on the average of three parallel sections through the optic nerve in each mouse, pathology was classified with regard to the number and extent of lesions, whether defined by staining or loss of staining: + denotes mild changes of limited extent, with multiple focal staining or loss thereof; ++ denotes more extensive changes by staining intensity or loss and cumulative/long; and +++ denotes either very strong staining or total loss of staining over an extensive cumulative area.

All analyses were done in a blinded fashion. Histopathological evaluation of optic nerves included loss of AQP4 and glial fibrillary acidic protein (GFAP), demyelination, and deposition of C. An anti-myelin oligodendrocyte glycoprotein (MOG) antibody [protein G affinity-purified supernatant from hybridoma clone Z2 (provided by Prof. Chris Linington, Glasgow University, UK)] was used to evaluate demyelination, and deposition of C was examined by an anti-C5b-9 (C9neo) staining [rabbit anti-C5b-9 (Abcam, UK)].

#### Quantitative RT-PCR

RNA was extracted using RNeasy Mini Kit according to the manufacturer's protocol (Qiagen, Germany). Reverse transcription was performed with M-MLV reverse transcriptase (Invitrogen, USA) according to the manufacturer's instructions. Quantitative real-time polymerase chain reaction (qPCR) was performed using 1 μl of cDNA combined with 24 μl of Maxima probe/ROX qPCR master mix (Thermo Fisher Scientific, Lithuania) with primer and probe sequences as follows: Interferon regulatory factor-7 (IRF7) (forward CACCCCCATCTTCGACTTCA, reverse CCAAAACCCAGGTAGATGGTGTA, probe CACTTTCTTCCGAGAACT MGB), CXCL10 (forward GCCGTCATTTTCTGCCTCAT, reverse GGCCCGTCATCGATATGG, probe GGACTCAAGGGATCC MGB), interleukin-6 (forward TATGAAGTTCCTCTCTGCAAGAGA, reverse TAGGGAAGGCCGTGGTT, probe CCAGCATCAGTCCCAAGAAGGCAACT), and IFN-β (forward GCGTTCCTGCTGTGCTTCTC, reverse TTGAAGTCCGCCCTGTAGGT, probe CGGAAATGTCAGGAGCT).

qPCRs were performed on a QuantStudio^TM^3 Real-Time PCR instrument (Applied Biosystems, Thermo Fisher Scientific, Denmark). Samples were run as triplicates. CT values were determined, and results were expressed relative to 18S rRNA (2^Δ*CT*^ method) as endogenous control (TaqMan^TM^ Ribosomal RNA control Reagents kit; Applied Biosystems, USA). cDNA was diluted 1/500 for 18S rRNA analysis.

#### Immunohistochemistry

Tissue sections were deparaffinized in xylene followed by a series of decreasing ethanol concentrations and finally rinsed in PBS. After this, disabling (by exhaustion) of endogenous peroxidase activity was performed by incubation in H_2_O_2_ (Sigma-Aldrich, Germany) in methanol (VWR International, France) for 10 min at room temperature followed by washing in deionized H_2_O. The target antigens were retrieved by microwaving the sections in TEG [6.055 g Tris base (Sigma-Aldrich T1378, USA), 0.950 g EGTA (Sigma-Aldrich, USA) in 5 L H_2_O, pH between 8.95 and 9.1] buffer with 0.05% Tween 20 (Sigma-Aldrich, USA) for 15 min. Sections were then blocked for endogenous biotin using Biotin Blocking system (DAKO, Agilent, Denmark) and washed in PBS with 0.2% Triton X-100 (Sigma-Aldrich, USA) (PBST).

Briefly, sections were washed in PBST followed by blocking for non-specific staining with 3% bovine serum albumin (BSA, Sigma-Aldrich, USA) in PBST for 30 min. Sections were incubated for 1.5 h with primary antibody [biotinylated anti-MOG (1:50)]. To verify antibody specificity, control sections were incubated with corresponding concentrations of unimmunized rabbit immunoglobulin fraction (DAKO Denmark A/S, Denmark).

The sections were then washed with PBST and incubated with streptavidin-horseradish peroxidase (1:200) (GE Healthcare, Little Chalfont, UK) at room temperature for 1 h. Sections were then washed with PBS, developed by adding 3,3′-diaminobenzidine [DAB, 0.5 mg/ml (Sigma-Aldrich, USA] and H_2_O_2_ (0.033%) for 2 min and washed twice in PBS. Finally, sections were dehydrated using increasing concentrations of ethanol, cleared in xylene, and mounted with DPX mounting medium (Merck KGaA, Germany).

#### Immunofluorescence

Tissue sections were deparaffinized, hydrated, and washed once in PBS or water; antigens were retrieved as for immunohistochemistry.

Sections that were to be stained with anti-AQP4 were blocked for non-specific staining similarly as for immunohistochemistry. Sections were incubated with primary antibody for 1–2 h [rabbit anti-AQP4 (1:500)]. To verify antibody specificity, sections were stained with similar concentrations of unimmunized rabbit immunoglobulin. Sections were washed in PBST and incubated for 1 h with primary and secondary antibodies [monoclonal mouse anti-GFAP Cy3 conjugated antibody (1:1,000) (C9205, Sigma-Aldrich, Merck, USA) and donkey anti-rabbit IgG Alexa Fluor 488 (1:200) (A21206, Invitrogen, Fisher Scientific), respectively]. Sections were then washed once in PBS and then for 5 min in PBS containing 300 nM 4′,6-diamidino-2-phenylindole (Molecular Probes, Invitrogen Detection Technologies, USA) (DAPI) to stain nuclei and mounted using Gelvatol [2 g glycerol (Sigma-Aldrich, USA), 1 g polyvinyl alcohol (Sigma-Aldrich, USA), and 7 ml PBS].

Sections stained with anti-C5b-9 were washed with TNT buffer (Fagron Nordic A/S, Copenhagen, Denmark) and blocked for non-specific staining with 2% BSA in Tris-buffered saline (TBS) for 10 min. Sections were incubated with primary antibody for 45 min [rabbit anti-C5b-9 (1:100)] and washed in TNT. Sections were then incubated for 45 min with the secondary antibody donkey anti-rabbit IgG Alexa Fluor 488 (1:200) and washed in TNT. The sections were blocked again with 2% BSA in TBS for 10 min followed by incubation for 1 h with the primary antibody monoclonal mouse anti-GFAP Cy3 conjugated antibody (1:2,000). The sections were then mounted with Vectashield Antifade Mounting Medium with DAPI (Vector Laboratories Inc., Burlingame, CA, USA).

#### Microscopy

Loss of AQP4 and GFAP, deposition of C, and demyelination were analyzed on full series (between 4 and 8 sections). Images were acquired using an Olympus DP73 digital camera mounted on an Olympus BX51 microscope (Olympus, Denmark). Images were processed using ImageJ software (1.51j8).

#### Statistical Analysis

Results, unless specified otherwise, were analyzed by two-tailed unpaired *t*-test using GraphPad Prism software (GraphPad Software Inc., USA). A *p* ≤ 0.05 was considered to be statistically significant. Data are presented as mean ± SEM.

## Results

### Intrathecal AQP4-IgG+C Induced Astrocytopathy in Optic Nerve

We previously reported that AQP4-IgG in CSF becomes widely distributed in the brain and causes C-dependent astrocyte injury in the brain stem, cerebellum, periventricular areas, and brain parenchyma (14, 15). To determine whether substances intrathecally injected into the CSF of mice can access the optic nerve, we used the tracer Evans blue in a diffusion study comparing with unmanipulated mice (Figure 1E). We demonstrated that the tracer reached the optic nerve of a mouse and was detectable around the optic nerve 0.5 h after intrathecal injection of the tracer into the CSF *via* cisterna magna (Figure 1F). The tracer thereafter diffused rapidly and was almost not apparent after 48 h (Figure 1G).

**Figure 2:**
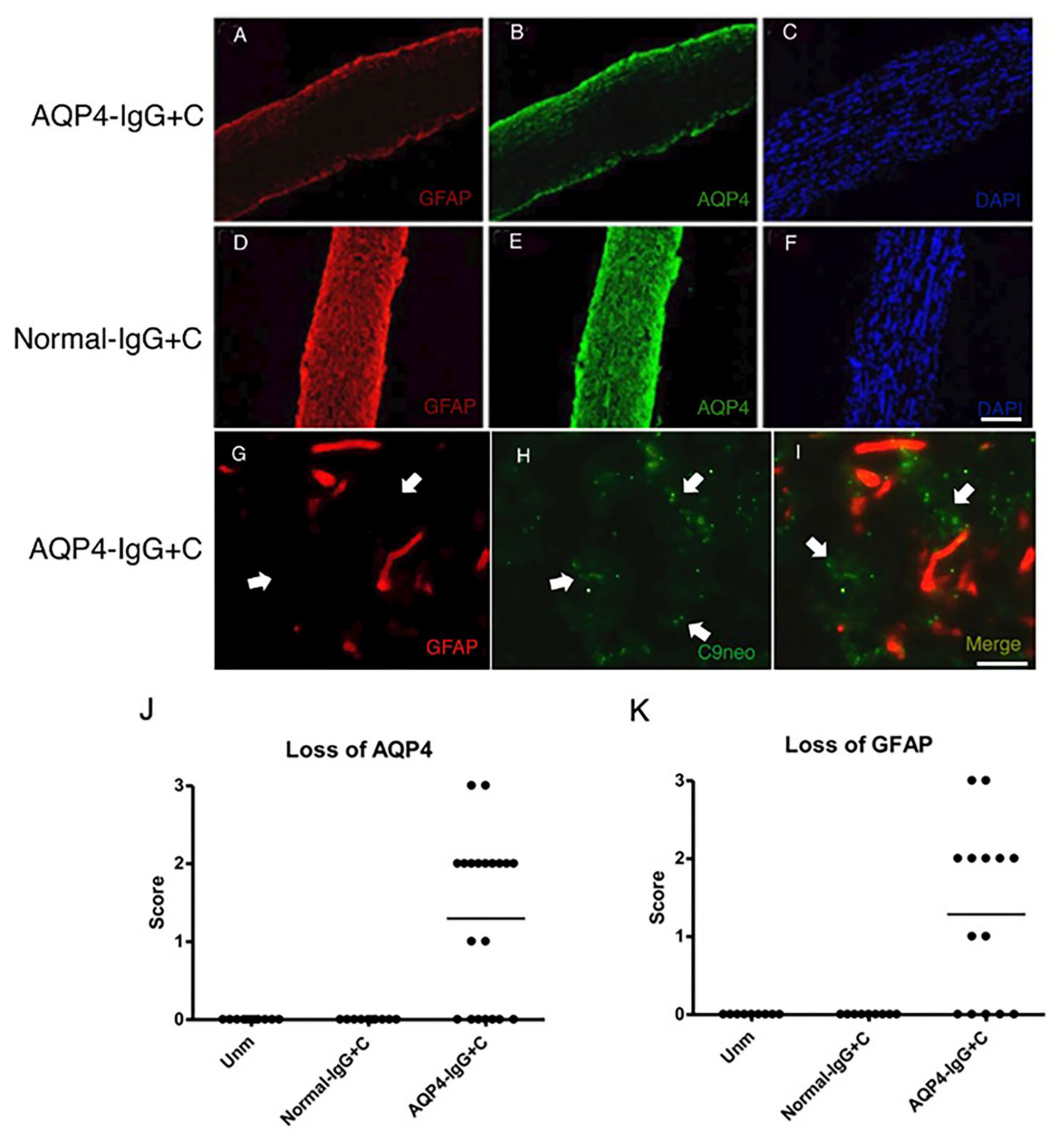
Aquaporin-4-immunoglobulin G (AQP4-IgG)-mediated optic nerve astrocytopathy. Micrographs show longitudinal sections of the optic nerve of animals that received intrathecal injections of AQP4-IgG + complement (C) (*n* = 14) **(A–C)** or normal-IgG+C (*n* = 9) **(D–F)** as control. Sections were stained for glial fibrillary acidic protein (GFAP) **(A,D)**, AQP4 **(B,E)**, and nuclei with 4',6-diamidino-2-phenylindole (DAPI) **(C,F)**. Two consecutive intrathecal injections of AQP4-IgG+C induced astrocyte pathology with loss of GFAP [**(A)** corresponds to severity: + + +] and AQP4 [**(B)** corresponds to severity: + + +] in optic nerves. Deposition of activated complement coincided with perivascular GFAP loss, both denoted by white arrows [**(G)**: GFAP staining and **H**: C9neo (anti-C5b-9) staining]. Optic nerve astrocytopathy was not seen in mice receiving normal-IgG+C **(D, E)**. Graphs showing semi quantitative score for loss of AQP4 **(J)** (unmanipulated, *n* = 11; normal-IgG+C, *n* = 10; and AQP4-IgG+C, *n* = 20) and GFAP **(K)** (unmanipulated, *n* = 9; normal-IgG+C, *n* = 9; and AQP4-IgG+C, *n* = 14) staining. Scale bar: 100 μm **(A–F)**, 20 mm **(G–I)**.

**Figure 3:**
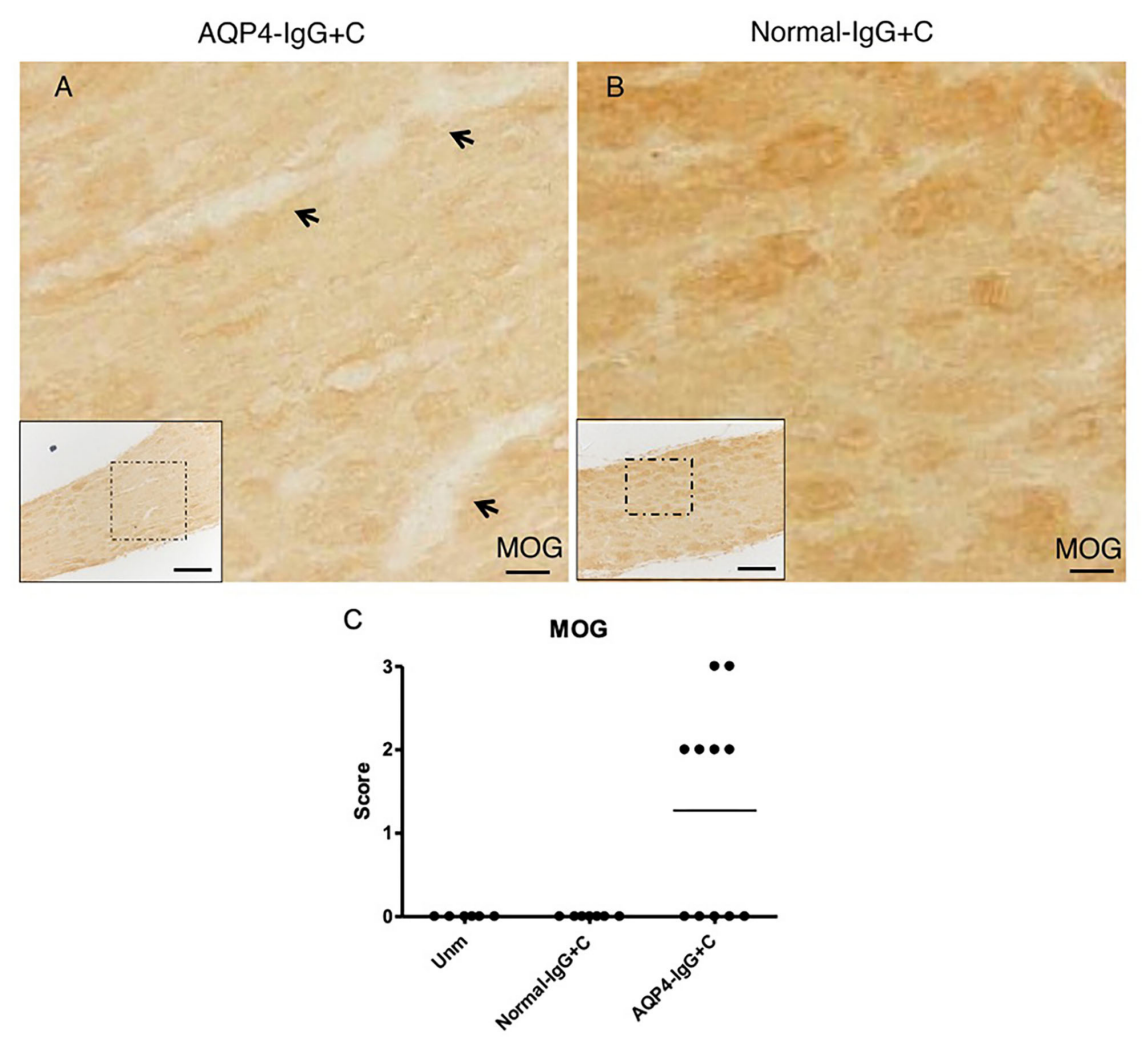
Pathogenic effect of aquaporin-4-immunoglobulin G+complement (AQP4-IgG+C) on optic nerve. Micrographs show longitudinal sections of the optic nerve of animals that received intrathecal injections of AQP4-IgG+C (*n* = 11) **(A)** or normal-IgG+C (*n* = 6) **(B)** as control. Sections were stained for myelin oligodendrocyte glycoprotein (MOG). Intrathecal injections of AQP4-IgG+C induced focal loss of MOG immunoreactivity, indicating focal demyelination [**(A)** corresponds to severity: ++] in optic nerves. Such pathology was not observed in mice receiving normal-IgG+C **(B)**. Graph showing semiquantitative score for loss of MOG staining **(C)** in mice receiving intrathecal AQP4-IgG+C (*n* = 11) or normal-IgG+C (*n* = 6). Additional control mice were unmanipulated wild-type (WT) mice (*n* = 6). Scale bar: 20 μm **(A,B)**, 100 μm (inserts).

We administered purified IgG from an AQP4-IgG-positive NMOSD-ON patient together with C to WT mice by intrathecal injection into the CSF *via* cisterna magna on two consecutive days. Histological analyses were performed 4 days after the first injection. Intrathecal injections of AQP4-IgG+C induced astrocyte pathology in optic nerves with loss of AQP4 (Figure 2B) in 13 out of 20 mice (65%) and loss of GFAP (Figure 2A) in nine out of 14 mice (64.3%). Deposition of activated C (C9neo staining) coincided with astrocyte pathology including perivascular localization of GFAP staining (Figures 2G–I; Supplementary Figure 1). We confirmed loss of AQP4 and GFAP immunoreactivities within the NMOSD-like lesions in the optic nerve and increased expression of GFAP around the lesions by single staining in parallel sections (DAB staining) (Supplementary Figures 2A,B). Additionally, we observed focal demyelination, illustrated by reduced MOG immunoreactivity (Figure 3A) in six out of 11 mice (55%). As expected, NMOSD-like pathology in the optic nerve was not seen in mice receiving normal human IgG and C (Figures 2D–F, 3B).

### Contribution of Type I Interferon Signaling in NMOSD-Like Pathology in Optic Nerve

**Figure 4:**
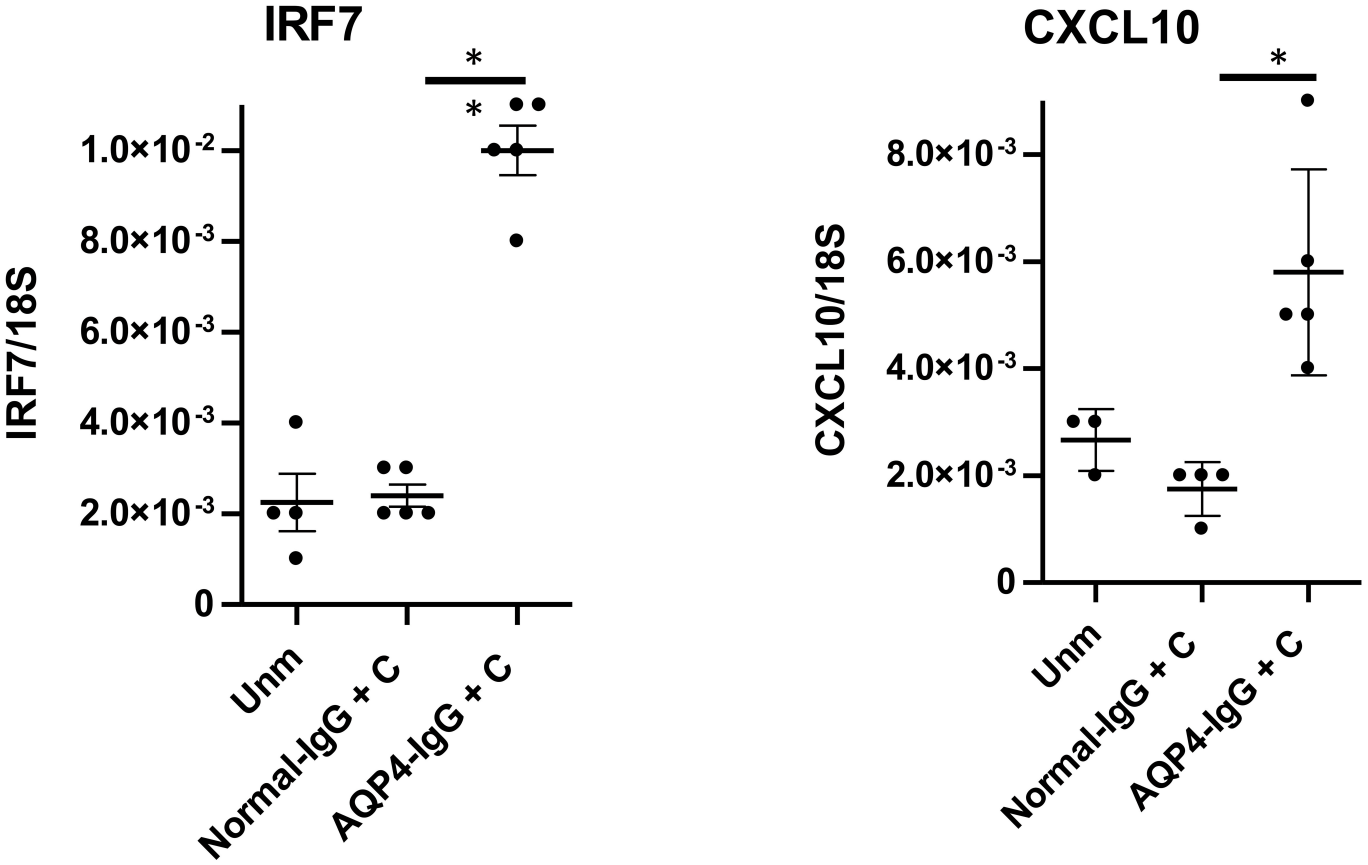
Upregulation of gene expression for interferon regulatory factor-7 (IRF7) and CXCL10 followed by intrathecal injections of aquaporin-4-immunoglobulin G + complement (AQP4-IgG+C) in wild-type (WT) mice. Graphs showing IRF7 and CXCL10 gene expression in unmanipulated WT mice and in WT mice that received intrathecal injections of normal normal-IgG+C or AQP4-IgG+C. Data were analyzed by two-tailed non parametric Student's *t* test followed by Mann-Whitney test. The data represent pooled results from two separate experiments. IRF7: unmanipulated (unm), *n* = 4; normal-IgG+C, *n* = 5; AQP4-IgG+C, *n* = 5. CXCL10: unmanipulated, *n* = 3; normal-IgG+C, *n* = 4; AQP4-IgG+C, *n* = 5. One unmanipulated mouse and one normal-IgG+C-injected mouse were removed, as they were determined to be outliers.

We have previously published that transfer of pathology in the brain with AQP4-IgG depends on type I IFN signaling (16, 17). We assessed induction of cytokines, chemokines, and transcription factors in the optic nerve by NMOSD-like pathology by performing RT-qPCR 2 days after the first of the two consecutive intrathecal injections of AQP4-IgG+C to WT mice to measure gene expression. This analysis showed a significant upregulation in gene expression of IRF7 and CXCL10, two canonical type I IFN signature genes (16, 17), in mice receiving AQP4–IgG+C compared to mice receiving normal-IgG+C (Figure 4). No difference was observed in gene expression of IFN-β or IL-6 in mice treated with AQP4-IgG+C compared to control mice.

**Figure 5:**
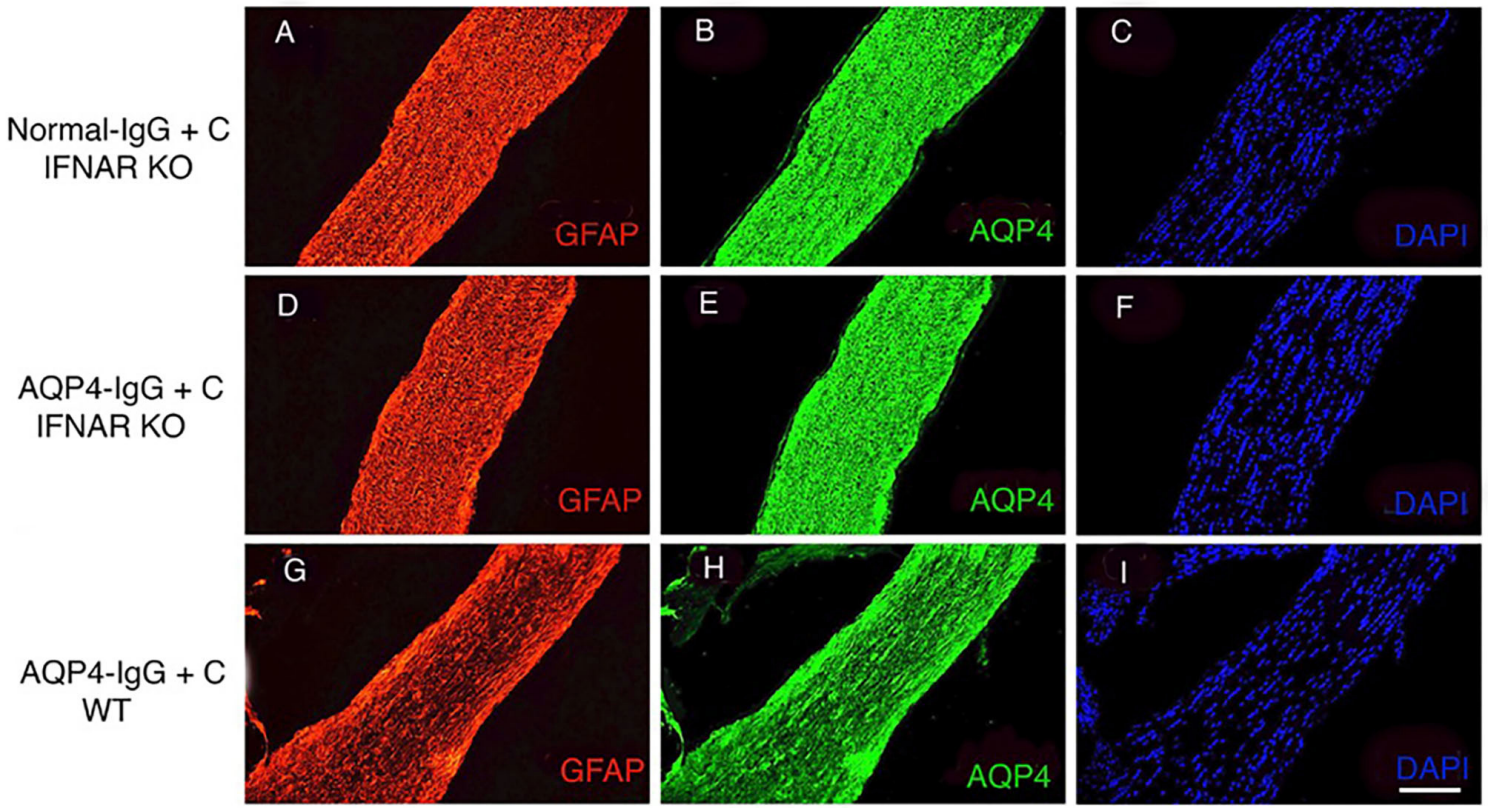
Lack of astrocytopathy in type I interferon receptor (IFNAR1)-deficient mice compared to wild-type (WT) mice. Micrographs show longitudinal sections of the optic nerve of IFNAR1-deficient mice that received intrathecal injections with normal-immunoglobulin G (IgG) + complement (C) (*n* = 3) **(A–C)** or aquaporin 4 (AQP4–IgG+C (*n* = 3) **(D–F)**. Controls included unmanipulated mice (*n* = 3) (data not shown). Additionally, WT mice received intrathecal injections of AQP4-IgG+C (*n* = 2) **(G–I)**. Sections were stained for glial fibrillary acidic protein (GFAP) **(A,D,G)**, AQP4 **(B,E,H)**, and 4′,6-diamidino-2-phenylindole (DAPI) **(C,F,I)**. Intrathecal injections of AQP4-IgG+C induced astrocyte pathology with loss of GFAP **(G)** corresponds to severity: + + + and AQP4 **(H)** corresponds to severity: + + + in optic nerves of WT mice. Due to long-segment astrocytopathy, the scoring level corresponds to degree + + +, and the intensity of loss is ++. Such pathology was not observed in IFNAR1-KO mice receiving normal-IgG+C **(A,B)** or AQP4-IgG+C **(D,E)**. Scale bar: 100 μm **(A–I)**.

Given that the expression of IRF7 was upregulated, we investigated the role of type I IFN signaling in NMOSD-like pathology in the optic nerve. We intrathecally injected AQP4-IgG+C into IFNAR1-KO mice and assessed histopathology at 4 days. NMOSD-like pathology was absent in IFNAR1-KO mice (Figures 5D,E) similar to controls, IFNAR1-KO mice injected with normal-IgG+C (Figures 5A,B). In contrast, pronounced loss of AQP4 and GFAP staining was observed in WT mice receiving AQP4-IgG+C (Figures 5G,H). The histopathology data confirm our previous observations on the lack of NMOSD pathology in the brain of IFNAR1-KO mice (17).

## Discussion

In the current study, the pathogenic effects of NMOSD patient-derived IgG, delivered *via* the intrathecal route, on the optic nerve have been assessed in a mouse model. We showed that AQP4-IgG together with C was sufficient to induce characteristic NMOSD-like pathology in the optic nerve with loss of AQP4 and GFAP, deposition of activated C, and demyelination in WT mice. The data also showed that intrathecal injection of AQP4-IgG with C induced upregulation of genes for the IFN signaling-associated pro-inflammatory chemokine CXCL10 as well as IRF7 in the optic nerve. Furthermore, we provide evidence for the requirement for type I IFN signaling in NMOSD-like pathology consistent with previous findings that deficiency of type I IFN signaling protects against astrocyte damage (17). This experimental model of antibody-mediated ON (NMOSD-ON) may be useful to clarify elements of pathogenesis and to pave the way for the development of new treatment strategies, as the optic nerve is a well-defined anatomical structure.

NMOSD is a severe autoimmune disease of the CNS, which is characterized by preferential effect on the optic nerve and spinal cord (21). Approximately 90% of NMOSD patients will have at least one episode of ON during the course of disease, and ON is frequently the first symptom of NMOSD (21, 23). High frequency of ON in NMOSD patients may be explained by elevated AQP4 expression in the optic nerve (24). Additionally, the optic nerve is heavily vascularized, and the blood–brain barrier (BBB) is very permissive in this area (25). Astrocytes are the primary target of the NMOSD-related pathogenic process (26). Clinically, astrocytic damage is reflected by elevated levels of soluble GFAP in CSF, distinguishing NMOSD from MS patients who show elevated levels of markers for myelin damage such as myelin basic protein in the CSF (6). Over the past two decades, a broadening of the clinical spectrum of ON in particular with regard to antibody-mediated ON has been appreciated, enabling the development of experimental models that may facilitate a mechanistic understanding.

Experimentally, NMOSD-ON has been mimicked *in vivo* by transfer of human AQP4-IgG to animals with pre-established experimental autoimmune encephalomyelitis (EAE) (27) or by direct intracerebral injection near the optic chiasma together with human C *via* continuous infusion using an implanted mini-pump (11). Here, we describe a model of antibody-mediated ON that produces pronounced astrocytopathy in the optic nerve that does not require simultaneous induction of EAE nor surgical trauma to the CNS. There is no confounding tissue inflammation that makes it a useful model for further understanding of mechanisms leading to optic nerve pathology, a hallmark of NMOSD. However, the astrocytopathy in the model described here is not restricted to the optic nerve, as we have previously demonstrated development of lesions in other parts of the CNS, including the brain, cerebellum, brain stem, and periventricular areas following intrathecal administration of AQP4-IgG (14). The incidence rate of astrocytopathy in the optic nerve (65%) is similar to that previously reported by us in other regions of the brain (14). The fact that not all injected animals develop pathology may be due to several reasons. Firstly, the variable efficiency of intrathecal injection of two single boluses of AQP4-IgG together with C into the CSF. Secondly, recirculation of the CSF that may lead to dilution of the AQP4-IgG infusion.

The development of astrocytopathy in our model requires injections of exogenous C as it does not occur in mice injected with AQP4-IgG alone (14). In contrast, it has been demonstrated in rat models that exogenous C is not necessary for development of lesions (28). This reflects the ability of human IgG to fix rat but not murine C (28). The clinical experience with inhibition of C pathway by anti-C5 (eculizumab) as standard therapy for NMOSD (29) suggests that C activation is important for the astrocyte damage in NMOSD. It is also supported by an experimental study showing more severe pathology in mice lacking the C inhibitor protein CD59 (11). In an interesting study (30) of microglia–astrocyte interaction in a spinal cord model for NMOSD, C-driven interaction *via* C3 between microglia and astrocytes was described. Those data support that early NMOSD-like immunohistopathology requires microglia/astrocyte crosstalk with a role for astrocyte-derived C3 acting *via* C3aR on microglia. The experimental design in that study involved continuous infusion of AQP4-IgG into the mouse spinal subarachnoid space *via* intrathecal catheter (30). Pathology was induced independently of exogenous (human) C; however, it did not extend to loss of astrocytes with classical C cascade, unlike in clinical NMOSD (31).

We observed significant upregulation of type I IFN-stimulated genes *IRF7* and *CXCL10* in the optic nerve in NMOSD-like pathology. Of note, CXCL10 was also shown to be upregulated in the CSF and sera from NMOSD patients (32). This is in line with our recent study showing a pronounced type I IFN transcriptomic footprint in the brain tissue of mice with NMOSD-like pathology (16). Moreover, we have shown that similarly to brain pathology (17), lesion formation in the optic nerve was dependent on type I IFN signaling, pathology being completely absent in mice lacking the receptor for type I IFN. Furthermore, clinical studies have shown an association between elevated levels of serum type I IFN (IFN-alpha) and development and activity of NMOSD (33, 34) as well as negative effect of IFN-β in NMOSD patients (35–39). Our recent study showed that microglia are the main responders to type I IFN in NMOSD-like pathology and that they are critical for the development of the disease (16). It is likely that they play a similar role in ON.

In conclusion, we describe the induction of ON in an animal model for NMOSD. By utilizing the intrathecal route for administration of autoantibody to the CSF, our model mimics human disease pathophysiology. Importantly, this animal model shows the involvement of the optic nerve in NMOSD and the contribution of type I IFN signaling in the disease process. This ON model as well as the approach used for isolation of the intact optic nerve may contribute to understanding of the immunopathogenesis of antibody-mediated ON (NMOSD-ON) diseases.

## Data Availability Statement

The original contributions presented in the study are included in the article/Supplementary Material, further inquiries can be directed to the last authors.

## Ethics Statement

The animal study was reviewed and approved by the Danish Animal Experiments Inspectorate (2020-15-0201-00652). The studies involving human participants were reviewed and approved by the Danish Regional Committee on Biomedical Research Ethics for the Region of Southern Denmark (ref. no. S-20080142). The patients/participants provided their written informed consent to participate in this study.

## Author Contributions

SFS: study design, acquisition of data, statistical analyses, interpretation of results, drafting, and revising the manuscript. MW, AW, STL, and MTM: acquisition of data, interpretation of results, and revising the manuscript. RK and DA: acquisition of data and revising the manuscript. TO: study design, acquisition of data, interpretation of results, and revising the manuscript. NA: study concept and design, acquisition of data, interpretation of results, revising manuscript, and approving final version. All authors contributed to the article and approved the submitted version.

## Conflict of Interest

The authors declare that the research was conducted in the absence of any commercial or financial relationships that could be construed as a potential conflict of interest.

## Publisher's Note

All claims expressed in this article are solely those of the authors and do not necessarily represent those of their affiliated organizations, or those of the publisher, the editors and the reviewers. Any product that may be evaluated in this article, or claim that may be made by its manufacturer, is not guaranteed or endorsed by the publisher.

## Abbreviations

AQP4-IgG: aquaporin-4-immunoglobulin G
C: complement
CSF: cerebrospinal fluid
IFN: interferon
IFNAR1-KO: type I interferon receptor-deficient
IRF7: interferon regulatory factor-7
GFAP: glial fibrillary acidic protein
NMOSD: neuromyelitis optica spectrum disorder
ON: optic neuritis
WT: wild-type.
