# Supplementary figures and images for: An Experimental Model of Neuromyelitis Optica Spectrum Disorder–Optic Neuritis: Insights Into Disease Mechanisms
Source: Front Neurol. 2021 Jul 23;12:703249. doi: 10.3389/fneur.2021.703249 (PMC8345107; doi:10.3389/fneur.2021.703249)

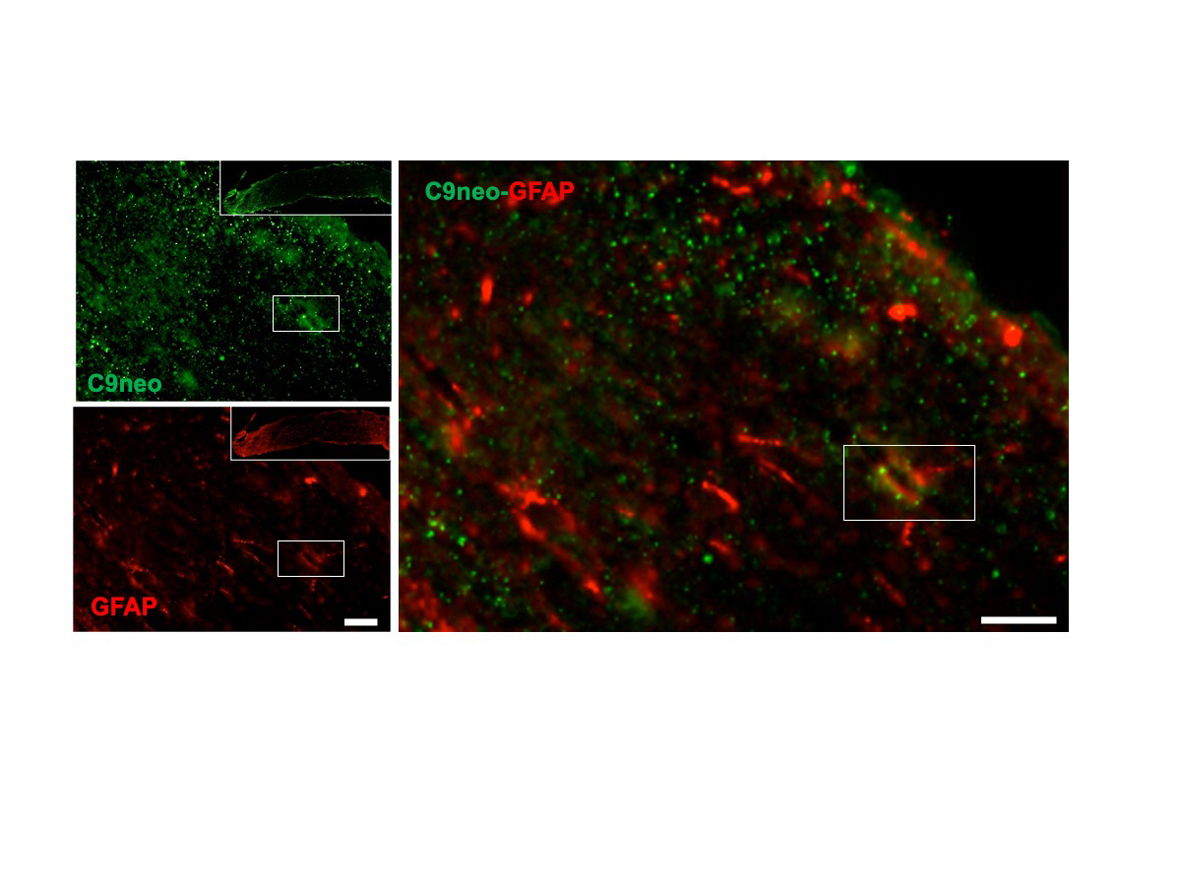

Supplement: Supplementary file 1 [file Image_1.JPEG]

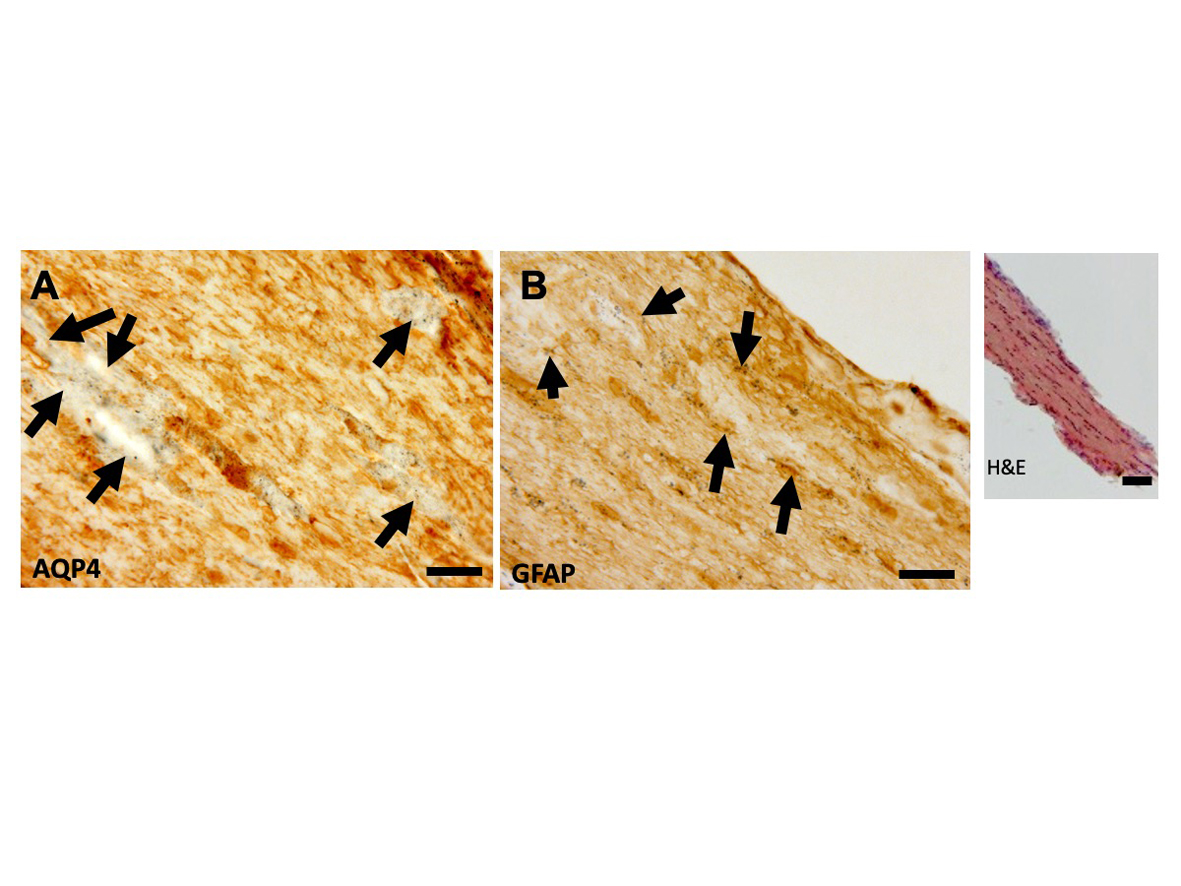

Supplement: Supplementary file 2 [file Image_2.JPEG]
